# Intrauterine Growth-Restricted Female Yucatan Miniature Pig Neonates Fed Parenteral Nutrition Exhibit Early Catch-Up Growth Leading to Obesity and Ectopic Fat Deposition in Adulthood

**DOI:** 10.1016/j.tjnut.2025.05.031

**Published:** 2025-05-27

**Authors:** Raniru S Randunu, Lee-Anne Huber, Janet A Brunton, Robert F Bertolo

**Affiliations:** 1 Department of Biochemistry, Memorial University of Newfoundland, St. John’s, Newfoundland and Labrador, Canada; 2 Department of Animal Biosciences, University of Guelph, Guelph, Ontario, Canada

**Keywords:** total parenteral nutrition, obesity, programming, ectopic fat, methyl nutrients, dyslipidemia, growth

## Abstract

**Background:**

Total parenteral nutrition (TPN) is often used as a lifesaving nutritional regimen in intrauterine growth-restricted (IUGR) neonates. However, nutrition perturbations during the early critical period may permanently program metabolism via methyl-dependent epigenetic alterations that can lead to obesity and dyslipidemia in adulthood. Methyl group availability can be increased by adding betaine and creatine to TPN.

**Objectives:**

We sought to determine whether TPN in early life would have long-term effects on the development of obesity, whether IUGR will exacerbate these TPN-induced effects, and whether supplementing betaine and creatine to TPN will alleviate these effects.

**Methods:**

Twenty four 7-d-old female piglets were randomly assigned to suckled, TPN-control diet (TPN-control), and TPN with betaine and creatine groups. Eight IUGR piglets were fed TPN (TPN-IUGR) as a fourth group. After 2 wk of TPN, all pigs received semi-restricted standard feed until adulthood (9 mo). Plasma and tissues were measured for lipids, hormones, and other metabolites associated with the development of obesity and metabolic syndrome.

**Results:**

Growth rates of TPN-IUGR were 32% and 42% greater than TPN-control during the 1–4 mo and 4–6 mo periods, respectively (*P* < 0.05), indicating catch-up growth, which led to greater adiposity, as indicated by a 16% higher backfat thickness at 9 mo (*P* < 0.05). Moreover, TPN-IUGR pigs had 2.54-fold and 3.28-fold greater (*P* < 0.05) accumulation of ectopic triglyceride deposition in the liver and skeletal muscle, respectively, possibly due to 67% greater fasting plasma nonesterified fatty acids (*P* < 0.001). TPN-control was not different from suckled or TPN with betaine and creatine pigs, suggesting that feeding TPN during the neonatal period did not result in obesity later in life, and methyl nutrient supplementation to TPN had no effect on obesity.

**Conclusions:**

IUGR has a profound effect on developing obesity later in life, but TPN feeding does not lead to obesity in adulthood.

## Introduction

Obesity is abnormal or excessive fat accumulation, which contributes to cardiovascular disease (CVD), dyslipidemia, liver steatosis, and metabolic syndrome [1]. Although genetic susceptibility and adult lifestyles are involved in these diseases, nutritional perturbations early in life could also increase risk of developing obesity later in life [1]. According to the developmental origins of health and disease theory, environmental exposures during early life can permanently influence health and vulnerability to disease in later life [2]. In this context, nutrition during the critical development window in early life plays an important role in programming diseases in adulthood. Total parenteral nutrition (TPN) provides essential nutrients intravenously when oral nutrition cannot be administered [3] and is prescribed frequently in preterm newborns, intrauterine growth-restricted (IUGR) neonates, and infants with certain medical problems that prohibit oral feeding. TPN support for neonates is associated with intestinal atrophy and bacterial translocation due to a damaged, underused, and malnourished gut [4], most likely predisposing them to altered metabolism, including the development of obesity later in life. TPN programs metabolism both acutely and permanently. Indeed, research has shown that feeding TPN to neonatal piglets leads to obesity [5], although contrary results were later reported suggesting these adverse metabolic phenotypes do not persist when piglets are examined into adolescence [6]. To our knowledge, no study has examined the long-term consequences of feeding TPN to neonates and the possibility of developing obesity as an adult.

Previous studies have illustrated that IUGR leads to a thrifty phenotype, resulting in catch-up growth and increasing risk of obesity in later life [7]. Both TPN and IUGR independently alter metabolism in the neonatal period [[8], [9], [10], [11], [12]]. Because IUGR neonates are likely to receive TPN in early life as a lifesaving feeding method, we hypothesized that the combination of TPN and IUGR will exacerbate changes in metabolism that lead to obesity in later life.

A growing body of evidence indicates that risk of developing chronic diseases is influenced by genetics and epigenetic factors that impact gene expression, such as DNA methylation [13,14]. Human and animal research demonstrates conclusively that early-life nutritional exposures can alter pre/postnatal development, leading to later obesity and obesity-related metabolic disorders, such as metabolic syndrome [15]. For example, maternal diets low in methyl donors led to hypomethylation of the *Agouti* gene [16], predisposing offspring to obesity [17]. Although there are many studies on the effects of perinatal methyl supply, including pregnancy and lactation, on metabolism [18], reports on the impact of only postnatal methyl donors on long-term body weight and metabolism changes are scarce. Appropriate epigenetics require adequate methyl groups, and epigenetic changes might result from methyl nutrient imbalances [19]. As the principal source of methyl groups, methionine is essential for numerous transmethylation reactions; however, methionine is not typically increased in the diet due to concerns about homocysteine toxicity, a risk factor for CVD [20]. Alternatively, betaine supplementation promotes methionine synthesis and decreases homocysteine amounts [21]. Recent research has demonstrated that most labile methyl groups are employed in synthesizing creatine and that enhancing the 1 methylation pathway affects the allocation of the limited methyl groups to other methylation pathways [22]. Therefore, if we augment the diet with creatine, the available methyl groups for DNA methylation will be enhanced. Thus, dietary betaine and creatine can increase methyl groups’ availability, with betaine increasing the remethylation of methionine and creatine, sparing methyl groups for other reactions. Notably, betaine and creatine are currently not included in commercial TPN products.

In this study, we hypothesized that the previously observed acute effects of TPN feeding in the early neonatal period on adiposity and dyslipidemia will persist into adulthood. Moreover, we hypothesized that adding betaine and creatine to TPN would mitigate these negative consequences of feeding TPN during the neonatal period. Finally, we hypothesized that IUGR will exacerbate obesity outcomes in adulthood that were induced by neonatal TPN feeding.

## Methods

### Experimental design and piglets

The Memorial University of Newfoundland Animal Care Committee approved the animal care and handling procedures in compliance with the Canadian Council on Animal Care guidelines. Thirty-two Yucatan miniature piglets, aged 7 d (study d 0), were obtained from the breeding colony at the Memorial University of Newfoundland for the study. The sample size was calculated using variance and difference estimates from previous studies by our laboratory in this model, using metabolic syndrome phenotype data as the primary outcomes [23,24]. As female pigs are more prone to develop metabolic syndrome [24], we utilized only female pigs in this study. The piglets were categorized into 4 treatment groups: *1*) TPN-control, consisting of normal birthweight piglets fed standard TPN; *2*) TPN – B + C, consisting of normal birthweight piglets fed TPN supplemented with betaine and creatine; *3*) TPN-IUGR, consisting of IUGR piglets fed standard TPN; and *4*) suckled (SowFed), consisting of normal birthweight suckling piglets. These 32 piglets were taken from 13 litters until *n* = 8 in each group. IUGR was defined as a piglet with a birth weight of ∼65% of the largest littermate [25]. The litters used were only from multiparous healthy sows (i.e., no gilts) with a litter size of ≥4 healthy piglets and were not overtly obese for the herd.

As previously described, all piglets underwent a surgical procedure to implant 2 venous catheters [26]. After surgery, intravenous trimethoprim (2.8 mg‧kg body weight^–1^) and sulfadoxine (1.4 mg‧kg body weight^–1^) (Borgal; Intervet Ltd) were given for 3 d postoperatively to control for potential infections. TPN-fed piglets were housed individually in metabolic cages equipped with a swivel and tether system (Lomir Biomedical) for 14 d. This setup allowed the piglets to move freely while receiving continuous intravenous diet infusion through the central vein catheter. The temperature in the piglet room was maintained at 27^°^C, and heat lamps were provided for additional heat. The lighting in the room followed a 12-h light-dark cycle. The SowFed piglets were returned to the sow after they recovered from surgery and were allowed to suckle until study day 14; all piglets resumed suckling within 2–3 h of being returned to the sow. After 14 d of treatment, the piglets underwent anesthesia and had their catheters removed. Following recovery, all piglets were weaned onto an ad libitum milk replacer (Grober Nutrition Inc) for 2–3 d, then transitioned to a standard grower diet, which they fed until the end of the study. Groups of 4 pigs were housed together for 8 mo and collectively fed 2% of their cumulative body weights, with adjustments made every 2 wk based on body weight measurements. Because Yucatan miniature pigs have the propensity to become obese when consumed ad libitum, we used restricted feeding (2% of body weight) during the grow-out phase, which is sufficient for normal growth and maintenance of Yucatan miniature pigs, as per our herd maintenance guidelines. Water was available ad libitum, and a 12-h day-night cycle was maintained with lights on from 08:00 to 20:00.

### Diet (TPN and grower)

The TPN diets were prepared following aseptic protocols [26], with modifications to the lipid source. The vitamins and minerals in the diets were provided at levels exceeding 100% of the estimated requirements for neonatal piglets [27]. Before infusion, each diet bag was supplemented with multivitamins (Multi12/K1 Pediatric; Baxter Corporation), iron dextran (Bimeda MTC Animal Health Inc), trace elements (Sigma-Aldrich Canada), and SMOFlipid™ emulsion (soybean oil, medium-chain triglyceride oil, olive oil, and fish oil) (Fresenius Kabi). We decided to use SMOFlipid as our lipid emulsion instead of intralipid because SMOFlipid 20% emulsion (mixture of soybean, medium-chain triglycerides (TGs), olive oil, and fish oil) is becoming the standard of care for many neonates. In the TPN – B + C group, betaine (235 mg‧kg body weight^–1^‧d^–1^) and creatine (118 mg‧kg body weight^–1^‧d^–1^) were added as supplements. Betaine was provided in a molar equivalent to the piglet’s methionine requirement [28], whereas creatine was supplemented to match the piglet’s creatine accretion rate [29]. The weight of the diet bags was regularly recorded to monitor the amount of diet infused. The complete diet provided 1.1 MJ of metabolizable energy·kg body weight ^–1^·d^–1^ with glucose (24.5 g·kg body weight^–1^·d^–1^) and lipid (20% SMOFlipid, Fresenius Kabi), each supplying half of nonprotein energy, with 15 g·kg body weight^–1^·d^–1^ of protein, supplied as free amino acids. The amino acid composition is shown in Supplemental Table 1. TPN was provided to the TPN groups at 12 mL·kg body weight^–1^·h^–1^.

After 14 d of dietary treatment and 2–3 d ad libitum milk replacer, piglets were weaned onto a grower pig diet (Supplemental Table 2; Eastern Farmers Co-op). This grower diet is the same 1 used in our previous grow-out study [23,24,30], providing 67% of energy from carbohydrates, 12% from fat, and 21% from protein.

### Monthly blood samples and body measurements

Body weight was measured daily from 7 to 21 d of age and every 2 wk during the grower phase of the study, which spanned from 1 to 9 mo. The feed conversion ratio was calculated by dividing each pig’s predicted feed intake (equivalent to 2% of body weight) by the weight they gained during specific time intervals (1–4 mo, 4–6 mo, and 6–8 mo). The fractional growth rate was determined by dividing the body weight gain during a given period by the initial body weight at the beginning of that time frame.

Monthly, blood samples were collected from the jugular vein of the pigs after an overnight fasting period. The blood was collected using EDTA tubes (Becton, Dickinson and Company). The collected samples were immediately centrifuged at 4000 × *g*; 15 min; 4°C to separate plasma. Lipid assays were conducted on fresh samples stored at 4°C, whereas the remaining plasma was stored at –80°C until further analysis [23].

### Necropsy

At 9 mo of age, the pigs were administered anesthesia using 105 mg‧kg^–1^ of sodium pentobarbital (Euthanyl; Bimeda MTC Animal Health Inc) and were mechanically ventilated during the collection of blood and organ samples to prevent tissue death from hypoxia. A cardiac puncture was performed to obtain blood samples, which were then placed in EDTA tubes. The blood samples were promptly centrifuged at 4000 × *g*; 15 min to separate the plasma. Using a freeze clamp, organs were removed, weighed, and rapidly frozen in liquid nitrogen. Both the organs and plasma samples were immediately stored at –80°C until further analysis. The thickness of the back fat was measured in the carcass at the midline of the back, just caudal to the last rib, to assess the amount of subcutaneous fat. The crown-to-rump length, chest circumference (caudal to the stifle joint), and abdominal girth (measured at the naval) were recorded at necropsy.

### Plasma lipid analysis

Fresh plasma was assayed for concentrations of plasma total cholesterol (TC), TG, HDL cholesterol, and free cholesterol using enzymatic assay kits (Sekisui Diagnostics PEI Ltd). Plasma non-HDL cholesterol was calculated by subtracting values of HDL cholesterol from TC. Plasma nonesterified fatty acids (NEFAs) were assayed using an enzymatic assay kit (Fujifilm Wako Diagnostics USA Corporation). Plasma from the necropsies was subjected to sequential density ultracentrifugation to isolate lipoprotein fractions [31]. After centrifuging necropsy plasma at 15,500 × *g*; 20 min; 12°C to remove chylomicrons, the supernatant was separated into VLDL, LDL, and HDL fractions. Enzymatic assay kits were used to determine the concentrations of TC and TG in these subfractions.

### Plasma adiponectin, leptin, glucose, insulin, and total bilirubin

Plasma leptin (catalog # LS-F22387; LifeSpan BioSciences, Inc) and adiponectin (catalog # LS-F21779; LifeSpan BioSciences, Inc) were assayed using ELISA. Plasma glucose concentrations were measured using an enzymatic assay kit (Sigma-Aldrich), and plasma insulin concentrations were measured using a Human Insulin ELISA kit (ab100578 Human Insulin ELISA kit; Abcam Inc). Plasma total bilirubin concentrations were measured using a colorimetric assay kit (catalog # MET-5010; Cell Biolabs, Inc).

### Liver phosphatidylcholine and phosphatidylethanolamine concentration

After lipids were extracted from the liver using the Folch method [32], phosphatidylcholine and phosphatidylethanolamine were isolated using thin-layer chromatography [22]. Liver phosphatidylcholine and phosphatidylethanolamine were determined by measuring total phosphate using a modified Bartlett method [33].

### Ectopic fat and cardiac muscle NEFA analysis

Lipids were extracted from known masses of the liver, skeletal muscle, and cardiac muscle samples [32]. The lipid-containing phase was evaporated to obtain dried lipids, which were dissolved in isopropanol and assayed for TG by enzymatic assay (Sekisui Diagnostic PEI Ltd). Cardiac muscle NEFA was assayed using an enzymatic assay kit.

### RNA extraction and real-time qPCR

Trizol was used to extract total RNA from liver tissues [34]. Genomic DNA contamination in RNA samples was removed using the DNAse enzyme (catalog # M610A; Promega). Using NanoDrop 2000, the concentration of extracted liver RNA samples was measured (Thermo Scientific). The integrity of each RNA sample was verified using 1.2% agarose gel. cDNA was produced from the extracted RNA samples using reverse transcription (catalog # A3500; Promega). Real-time qPCR primers (acetyl-CoA carboxylase (ACC-1), fatty acid synthase (FASN), sterol CoA desaturase, diacylglycerol acyltransferase -2 (DGAT-2), sterol regulatory element binding protein 1C, carnitine palmitoyltransferase 1), fatty acid translocase, β-actin; glyceraldehyde 3-phosphate dehydrogenase) were taken from previously published articles (Supplemental Table 3). The primers were also verified employing NCBI primer blast (www.ncbi.nlm.nih.gov/tools/primerblast) and obtained from Integrated DNA Technologies. The forward and reverse sequences of each pair of primers are listed (Supplemental Table 3). SYBR Green Supermix (catalog # 1708882; Bio-Rad) was used to begin qPCR amplification, and samples were processed on a mastercycler ep realplex system. The delta Ct values for the genes of interest and the reference genes were then calculated. β-actin and glyceraldehyde 3-phosphate dehydrogenase were both used as reference genes to normalize the gene expressions of interested genes, accounting for their primer efficiencies following the Vandesompele method [35].

### Plasma amino acid analysis

Reverse-phase HPLC was used to detect plasma amino acid concentration with the C18 column (Waters Corporation), as a method described elsewhere, after plasma derivatization of plasma with phenylisothiocyanate [36].

### Measurement of lipid peroxidation and antioxidant capacity

Plasma and liver thiobarbituric acid reactive substances were determined by measuring the concentration of malondialdehyde [37], with minor modifications as described for liver homogenates [38]. Liver antioxidant capacity was measured by assaying SOD (superoxide dismutase) (ab65354 SOD activity assay kit; Abcam Inc).

### Plasma metabolites

Concentrations of plasma betaine, choline, and dimethylglycine (DMG) concentrations were quantified by HPLC (Waters Alliance 2795; Waters Corporation; Atlantis HILIC (hydrophilic interaction liquid chromatography) silica 3 *μ*m 2.1 × 100 mm column) with a tandem mass spectrometer (micromass ultima triple-quad tandem mass spectrometer; Waters Corporation), as previously described [39,40]. Multiple-reaction monitoring mode was used to detect the compounds with the following transitions: [^2^H_11_] betaine 129➔68, betaine 118➔59, [^2^H_9_ methyl] choline 113➔69, choline 104➔60, DMG 104➔58. Plasma concentrations were calculated using calibration standards made using dialyzed plasma spiked with betaine, choline, and DMG. [^2^H_9_-methyl] choline and [^2^H_11_] betaine were used as internal standards [39]. Final concentrations of betaine, choline, and DMG were calculated using MassLynx Software (Waters Corporation).

Plasma total homocysteine concentrations were determined using reverse-phase HPLC and fluorescence detection of ammonium 7-fluoro 2-oxa-1,3-diazole-4- sulfonate thiol adducts [41]. Plasma creatine concentrations were measured by HPLC [42].

### Statistical analyses

The experimental groups were compared using a 1-way analysis of variance, and the group differences were determined using Dunnett’s post hoc test (GraphPad Prism 8.0; GraphPad Software), with the TPN-control assigned as the control group. The results are presented as means ± SD. Pearson’s correlation was used to compare the relationship between subcutaneous fat thickness and various parameters such as body growth measurements, plasma LDL, and non-LDL cholesterol at necropsy. The experimental groups were compared for monthly lipid parameters using a 2-way analysis of variance, with lipid parameters and time as the main effects (GraphPad Prism 8.0; GraphPad Software). If *P* < 0.05, differences were considered significant. Trends were noted if *P* = 0.05 − 0.15.

The feed conversion ratio was calculated by dividing each pig’s predicted feed intake (2% of body weight) by the body weight gain during each phase (1–4 mo/4–6 mo/6–8 mo). The fractional growth rate was calculated by dividing the gain in body weight for a given period by the initial body weight of that period.

## Results

### Growth and feed intake

**FIGURE 1:**
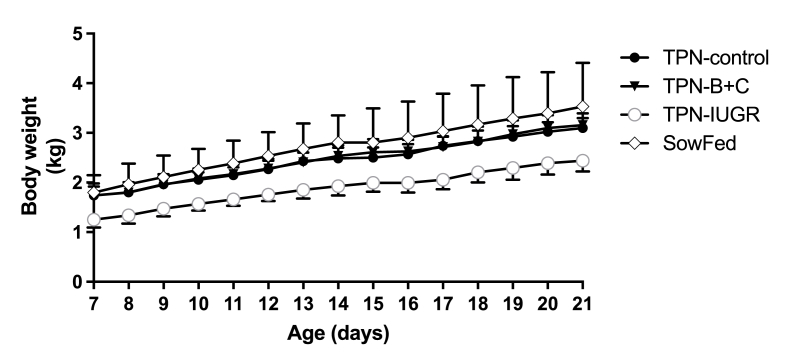
Body weights in TPN-control, TPN – B + C, TPN-IUGR, and SowFed Yucatan miniature pigs during the TPN feeding phase from 7 d to 21 d. Each symbol represents the mean ± SD; *n* = 8. SowFed, suckled; TPN, total parenteral nutrition; TPN – B + C, total parenteral nutrition with betaine and creatine; TPN-control, total parenteral nutrition control diet; TPN-IUGR, intrauterine growth-restricted piglets fed total parenteral nutrition control diet.

**FIGURE 2:**
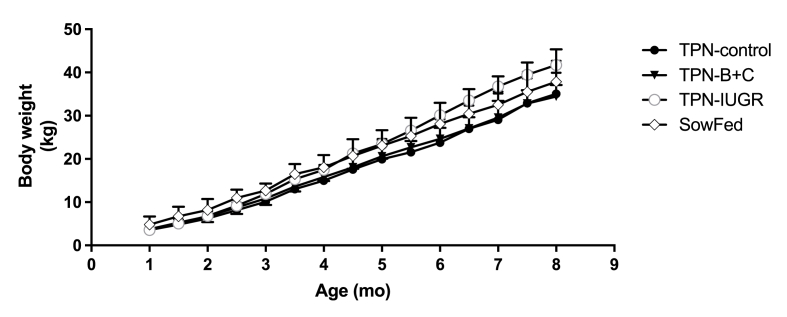
Body weights in TPN-control, TPN – B + C, TPN-IUGR, and SowFed Yucatan miniature pigs during the grow-out phase from 1 mo to 8 mo. Each symbol represents the mean ± SD; *n* = 8. SowFed, suckled; TPN, total parenteral nutrition; TPN – B + C, total parenteral nutrition with betaine and creatine; TPN-control, total parenteral nutrition control diet; TPN-IUGR, intrauterine growth-restricted piglets fed total parenteral nutrition control diet.

Because puberty in Yucatan miniature pigs occurs around 4 mo, we divided the growth parameters into the development phases shown in Table 1. IUGR pigs were selected to have lower body weights than control pigs at birth, and the body weights were significantly lower than control pigs at the start of the experiment. The lower body weight of the IUGR pigs remained significantly lower at the end of TPN feeding compared to the control pigs (i.e., 21 d); however, by 1 mo of age, the body weights of TPN-IUGR and TPN-control pigs were similar, suggesting IUGR pigs experienced “catch-up” growth (Table 1, FIGURE 1, FIGURE 2). After 1 mo, the body weights of all pigs in the presexual maturity, sexual maturity, and postsexual maturity phases did not differ between the groups until the end of the experimental period (9 mo). Body growth rates did not differ between groups during the TPN phase (7–21 d) (Figure 1); however, the growth rates of TPN-IUGR were higher during the presexual maturity and sexual maturity phases (1–4 mo and 4–6 mo, respectively) compared to TPN-control pigs (Table 1, Figure 2). At 9 mo, the experimental groups had no significant differences in body measurements.

**TABLE 1:** Growth parameters of Yucatan miniature pigs fed 4 experimental diets, TPN-control TPN-B+C, TPN-IUGR, and SowFed, during the neonatal period.

Age	TPN-control	TPN – B + C	TPN-IUGR	SowFed	TPN-control: SowFed ratio	TPN – B + C: SowFed ratio	TPN-IUGR: SowFed ratio
**Bodyweight (kg)**							
Birth	0.98 ± 0.17	1.02 ± 0.12	0.67 ± 0.10∗∗∗	1.01 ± 0.15	0.97	1.00	0.67
TPN start day (7 d)	1.74 ± 0.18	1.74 ± 0.25	1.25 ± 0.16∗∗	1.80 ± 0.35	0.97	0.97	0.70
TPN end day (21 d)	3.10 ± 0.20	3.15 ± 0.24	2.44 ± 0.21∗	3.53 ± 0.88	0.88	0.89	0.69
1 mo	3.54 ± 0.88	3.77 ± 0.61	3.57 ± 1.09	4.81 ± 1.87	0.74	0.78	0.74
4 mo	14.99 ± 3.29	15.83 ± 2.77	17.46 ± 2.54	18.06 ± 2.86	0.83	0.88	0.97
8 mo	35.05 ± 7.62	34.43 ± 5.49	41.74 ± 4.63	37.87 ± 7.49	0.93	0.91	1.13
**Body weight growth rate (g‧ d**^**–1**^**)**							
7–21 d	97.1 ± 6.2	101.1 ± 7.5	83.0 ± 8.5	117.9 ± 48.7	0.82	0.86	0.71
1–4 mo	130.1 ± 38.5	135.6 ± 30.9	171.8 ± 25.1∗	151.8 ± 31.9	0.86	0.89	1.13
4–6 mo	143.7 ± 52.5	148.5 ± 45.4	203.7 ± 28.7∗	165.1 ± 57.1	0.87	0.90	1.23
6–8 mo	190.2 ± 39.9	169.0 ± 34.9	194.8 ± 51.0	164.0 ± 59.3	1.16	1.03	1.19
**Fractional growth rate (g.kg body weight**^**–1**^**‧d**^**–1**^**)**							
7–21 d	40.1 ± 3.1	41.6 ± 5.1	47.6 ± 4.3	45.4 ± 11.7	0.88	0.92	1.05
1–4 mo	13.9 ± 2.7	13.7 ± 2.1	15.2 ± 3.3	13.0 ± 2.6	1.07	1.06	1.18
4–6 mo	7.3 ± 1.6	7.3 ± 1.7	8.5 ± 1.3	6.4 ± 2.6	1.14	1.13	1.33
6–8 mo	6.1 ± 0.6	5.3 ± 0.6	5.6 ± 1.2	5.0 ± 1.1	1.23	1.06	1.13
**Body measurements at 9 mo**							
Crown-to-rump length (cm)	112.1 ± 6.1	111.8 ± 6.3	118.1 ± 3.2	116.3 ± 6.9	0.96	0.96	1.02
Abdominal circumference (cm)	84.7 ± 6.8	82.8 ± 6.8	88.3 ± 4.1	84.4 ± 7.2	1.00	0.98	1.05
Chest girth (cm)	79.4 ± 4.3	79.3 ± 3.7	82.9 ± 4.3	77.7 ± 4.0	1.02	1.02	1.07
Subcutaneous fat thickness (mm)	43.2 ± 5.5	43.7 ± 3.8	50.0 ± 5.0∗	45.1 ± 5.7	0.97	0.98	1.12
**Feed conversion ratio**							
1–4 mo	1.56 ± 0.20	1.56 ± 0.12	1.47 ± 0.26	1.74 ± 0.23	0.89	0.89	0.84
4–6 mo	3.30 ± 0.98	3.45 ± 1.14	2.59 ± 0.49	3.43 ± 1.15	0.96	1.01	0.76
6–8 mo	3.83 ± 0.64	4.51 ± 1.20	4.55 ± 1.32	5.45 ± 2.13	0.70	0.83	0.84

Values are means ± SD; *n* = 7–8.

Abbreviations: ANOVA, analysis of variance; SD, standard deviation; SowFed, suckled; TPN, total parenteral nutrition; TPN – B + C, total parenteral nutrition with betaine and creatine; TPN-control, total parenteral nutrition control diet; TPN-IUGR, intrauterine growth-restricted piglets fed total parenteral nutrition control diet.

∗*P* < 0.05, ∗∗*P* < 0.001, ∗∗∗*P* < 0.0001; 1-way ANOVA with Dunnett’s post hoc test comparing to TPN-control. Ratios represent the means of each group divided by SowFed means within each row.

### Organ weights

Relative kidney, heart, and brain weights were lower in the TPN-IUGR pigs compared to TPN-control pigs as young adults (Table 2). In contrast, TPN-IUGR pigs had an increased subcutaneous fat thickness compared to TPN-control pigs at 9 mo (Table 2).

**TABLE 2:** Relative tissue weights and subcutaneous fat thickness at 9 mo in Yucatan miniature pigs fed 4 experimental diets during the neonatal period: TPN-control, TPN-B+C, TPN-IUGR, and SowFed.

Tissue/kg body weight	TPN-control	TPN – B + C	TPN-IUGR	SowFed	*P* value
Liver	17.80 ± 2.60	17.19 ± 2.37	15.58 ± 1.47	16.83 ± 1.99	0.235
Kidneys	3.39 ± 0.54	3.05 ± 0.34	2.63 ± 0.44∗	2.88 ± 0.20	0.008
Heart	4.10 ± 0.46	4.03 ± 0.57	3.37 ± 0.44∗	3.85 ± 0.37	0.026
Pancreas	1.33 ± 0.44	1.50 ± 0.39	1.29 ± 0.17	1.37 ± 0.38	0.691
Stomach	8.41 ± 1.12	8.27 ± 0.75	7.11 ± 2.62	8.95 ± 2.01	0.307
Brain	1.93 ± 0.25	1.92 ± 0.27	1.63 ± 0.17∗	1.75 ± 0.20	0.035
Subcutaneous fat (mm)	43.25 ± 5.52	43.75 ± 3.81	50.00 ± 5.01∗	44.33 ± 5.68	0.043

Values are means ± SD, *n* = 6–8.

Abbreviations: ANOVA, analysis of variance; SowFed, suckled; TPN, total parenteral nutrition; TPN – B + C, total parenteral nutrition with betaine and creatine; TPN-control, total parenteral nutrition control diet; TPN-IUGR, intrauterine growth-restricted piglets fed total parenteral nutrition control diet.

∗*P* < 0.05; 1-way ANOVA with Dunnett’s post hoc test comparing to TPN-control.

Liver TG content was greater in TPN-IUGR pigs than in TPN-control pigs at 9 mo; however, liver cholesterol content did not differ between the groups (Table 3). Plasma non-HDL cholesterol and NEFA concentrations were greater in TPN-IUGR pigs compared to TPN-control pigs; however, cardiac tissue NEFA concentration did not differ between the groups (Table 3). VLDL-TG and LDL cholesterol contents were greater, whereas VLDL cholesterol content was lower in TPN-IUGR pigs than in TPN-control pigs (Table 3). Plasma thiobarbituric acid reactive substances concentration was greater in TPN-control pigs compared to SowFed control at 9 mo as young adult pigs (Table 3). Fasted plasma glucose and insulin concentrations were not different in any experimental groups compared to the TPN-control group (Table 3).

**TABLE 3:** Tissue and plasma lipid profiles, lipid peroxidation, and antioxidant capacity of 9 mo old Yucatan miniature pigs fed a TPN-control, TPN-B+C, control TPN-IUGR and SowFed experimental diets during the neonatal period.

	TPN-control	TPN – B + C	TPN-IUGR	SowFed	P value
**Liver**					
Total cholesterol (mmol‧ g protein^–1^)	9.39 ± 2.53	9.39 ± 2.32	11.16 ± 2.54	8.60 ± 4.07	0.379
Total triglyceride (mmol‧ g protein^–1^)	3.77 ± 2.94	4.13 ± 2.58	9.60 ± 5.73∗	4.67 ± 4.37	0.036
PC (mmol‧ g protein^–1^)	0.66 ± 0.12	0.61 ± 0.12	0.68 ± 0.12	0.68 ± 0.13	0.626
PE (mmol‧ g protein^–1^)	0.43 ± 0.06	0.43 ± 0.07	0.47 ± 0.03	0.46 ± 0.06	0.413
PC/PE	1.53 ± 0.22	1.42 ± 0.16	1.57 ± 0.16	1.55 ± 0.35	0.625
SOD activity (inhibition rate %)	101.00 ± 3.36	101.55 ± 3.33	104.77 ± 3.05	102.07 ± 5.25	0.267
TBARS (μmol‧ g protein^–1^)	4.31 ± 0.83	5.06 ± 0.86	4.79 ± 1.10	4.77 ± 1.45	0.647
**Cardiac tissue**					
NEFA (mmol‧ g protein^–1^)	0.26 ± 0.04	0.30 ± 0.05	0.25 ± 0.06	0.31 ± 0.07	0.178
**Plasma**					
Total cholesterol (mmol‧ L^–1^)	1.405 ± 0.268	1.270 ± 0.216	1.617 ± 0.399	1.453 ± 0.117	0.140
Total HDL cholesterol (mmol‧ L^–1^)	0.974 ± 0.189	0.958 ± 0.191	0.867 ± 0.240	0.938 ± 0.067	0.665
Total non-HDL cholesterol (mmol‧ L^–1^)	0.460 ± 0.190	0.302 ± 0.095	0.750 ± 0.321∗	0.529 ± 0.114	0.005
Plasma-free cholesterol (mmol‧ L^–1^)	0.608 ± 0.174	0.484 ± 0.249	0.507 ± 0.104	0.536 ± 0.082	0.526
Plasma NEFA (mmol‧ L^–1^)	0.09 ± 0.04	0.09 ± 0.04	0.15 ± 0.03∗	0.08 ± 0.04	0.007
Total triglycerides (mmol‧ L^–1^)	0.397 ± 0.110	0.404 ± 0.113	0.422 ± 0.147	0.358 ± 0.106	0.752
TBARS (μmol‧ L^–1^)	6.52 ± 2.28	4.89 ± 0.93	5.31 ± 0.52	4.27 ± 0.99∗	0.018
Glucose (mmol‧L^–1^)	6.7 ± 1.7	7.7 ± 1.1	7.6 ± 1.4	7.6 ± 0.98	0.337
Insulin (μU‧mL^–1^)	58.3 ± 51.3	55.5 ± 35.0	74.2 ±52.8	84.3 ±52.5	0.654
**Plasma lipoprotein subfractions**					
Cholesterol					
VLDL (mmol‧ g protein ^–1^)	1.562 ± 0.656	1.427 ± 0.491	0.883 ± 0.263∗	1.421 ± 0.335	0.049
LDL (mmol‧ g protein ^–1^)	2.284 ± 0.551	2.390 ± 0.727	4.483 ± 2.062∗	2.640 ± 0.649	0.002
HDL (mmol‧ g protein ^–1^)	0.946 ± 0.364	0.957 ± 0.489	1.438 ± 0.496	0.762 ± 0.461	0.061
Triglycerides					
VLDL (mmol‧ g protein ^–1^)	2.125 ± 1.289	2.522 ± 0.863	3.492 ± 1.208∗	2.127 ± 0.873	0.054
LDL (mmol‧ g protein ^–1^)	0.364 ± 0.222	0.339 ± 0.148	0.254 ± 0.085	0.307 ± 0.209	0.667
HDL (mmol‧ g protein ^–1^)	0.025 ± 0.015	0.031 ± 0.018	0.020 ± 0.004	0.018 ± 0.013	0.235

Values are means ± SD, n = 6–8.

Abbreviations: ANOVA, analysis of variance; NEFA, nonesterified fatty acid; PC, phosphatidylcholine; PE, phosphatidylethanolamine; SOD, oxide dismutase; SowFed, suckled; TBARS, thiobarbituric acid reactive substance; TPN – B + C, total parenteral nutrition with betaine and creatine; TPN-control, total parenteral nutrition control diet; TPN-IUGR, intrauterine growth-restricted piglets fed total parenteral nutrition control diet.

∗P < 0.05; 1-way ANOVA with Dunnett’s post hoc test comparing to TPN-control.

**FIGURE 3:**
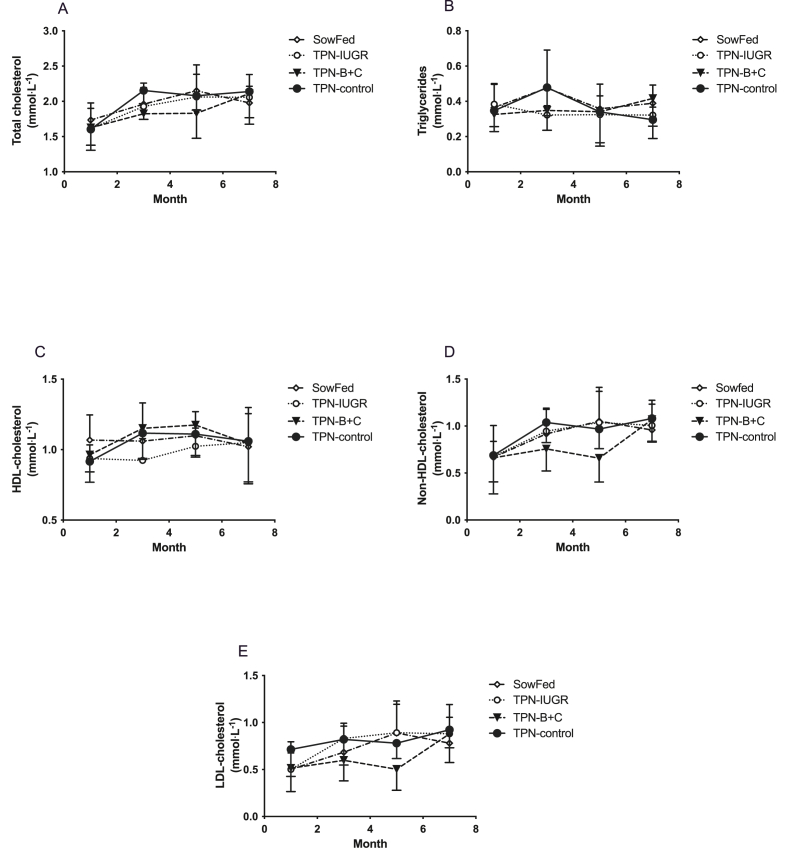
Monthly fasting plasma total cholesterol (A), triglycerides (B), HDL cholesterol (C), non-HDL cholesterol (D), and LDL cholesterol (E) concentrations in Yucatan miniature pigs fed TPN-control, TPN – B + C, TPN-IUGR, and SowFed during the neonatal period. Two-way ANOVA showed no treatment nor time effect for any of the parameters (*P* > 0.05). Values are mean ± SD; *n* = 7–8. ANOVA, analysis of variance; SowFed, suckled; TPN, total parenteral nutrition; TPN – B + C, total parenteral nutrition with betaine and creatine; TPN-control, total parenteral nutrition control diet; TPN-IUGR, intrauterine growth-restricted piglets fed total parenteral nutrition control diet.

Monthly plasma lipid parameters did not significantly differ between the groups at 1 mo, 3 mo, 5 mo, and 7 mo (Figure 3). However, regardless of the birth weight and feeding route in the neonatal period, TC, non-HDL cholesterol, LDL cholesterol, and HDL cholesterol increased with age in all groups.

**FIGURE 4:**
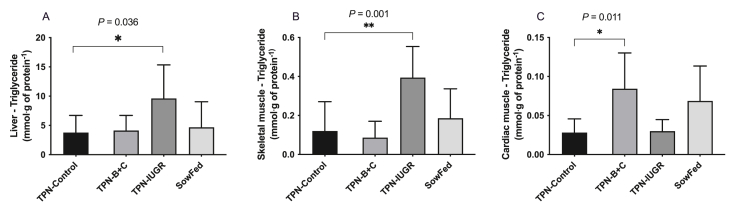
Liver (A), skeletal muscle (B), and cardiac muscle (C) triglycerides concentrations of adult Yucatan miniature pigs at 9 mo. Values are means ± SD; *n* = 6–8. Data were analyzed using 1-way ANOVA with Dunnett’s post hoc test comparing to TPN-control. ∗*P* < 0.05, ∗∗*P* < 0.001. ANOVA, analysis of variance; SowFed, suckled; TPN, total parenteral nutrition; TPN – B + C, total parenteral nutrition with betaine and creatine; TPN-control, total parenteral nutrition control diet; TPN-IUGR, intrauterine growth-restricted piglets fed total parenteral nutrition control diet.

The liver and skeletal muscle TG contents were greater in TPN-IUGR pigs compared to TPN-control pigs at 9 mo (Figure 4). Interestingly, TPN – B + C pigs had greater cardiac TG concentrations compared to TPN-control pigs, whereas the cardiac TG content of TPN-IUGR pigs was not different from that of TPN-control pigs (Figure 4C). However, the absolute concentration of TG in the cardiac muscle was relatively low compared to the TG concentration in the liver and skeletal muscle.

**FIGURE 5:**
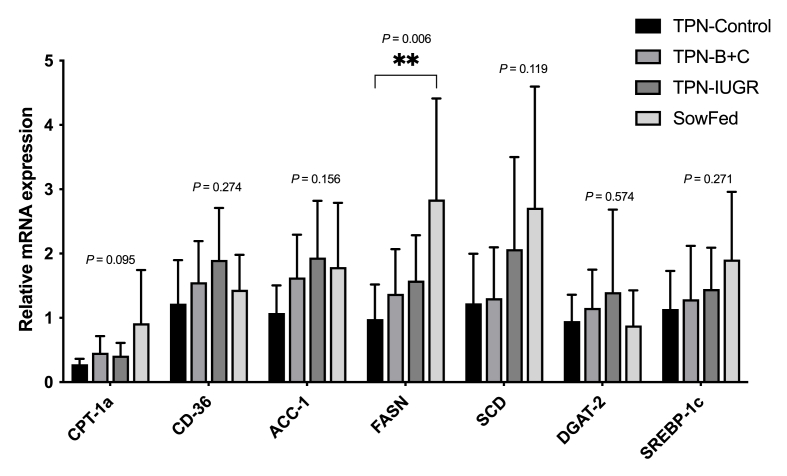
Relative mRNA expression of lipogenic genes in the liver of adult Yucatan miniature pigs (9 mo) fed 4 experimental diets. Data are means ± SD, *n* = 6–8 pigs. Data were analyzed using 1-way ANOVA with Dunnett’s post hoc test comparing to TPN-control. Geometric averaging of both β-actin and GAPDH was used to normalize the expression levels of genes of interest, and the data was corrected for primer efficiency using Vandesompele et al. [35]. ACC-1, acetyl-CoA carboxylase; ANOVA, analysis of variance; CD-36, fatty acid translocase; CPT1, carnitine palmitoyl transferase I; DGAT-2, diacylglycerol acyltransferase; FASN, fatty acid synthase; GAPDH, glyceraldehyde-3-phosphate dehydrogenase; mRNA, messenger ribonucleic acid; SCD, sterol coA desaturase; SowFed, suckled; SREBP-1C, sterol regulatory element binding protein 1C; TPN, total parenteral nutrition; TPN – B + C, total parenteral nutrition with betaine and creatine; TPN-control, total parenteral nutrition control diet; TPN-IUGR, intrauterine growth-restricted piglets fed total parenteral nutrition control diet.

Expression of the key genes involved in lipogenesis in the liver was not different between the experimental groups, except for FASN (Figure 5 [35]). SowFed pigs had a greater abundance of FASN mRNA in the liver compared to TPN-control pigs at 9 mo. Fatty acid translocase binds to long-chain free fatty acids and facilitates their transport to liver cells [43]; carnitine palmitoyltransferase 1a is active on the outer surface of mitochondria and serves as a regulatory site for fatty acid oxidation [44]; ACC-1 and FASN genes are responsible for the rate-limiting enzymes of de novo fatty acid synthesis [45,46]; sterol CoA desaturase is the gene responsible for the rate-limiting enzyme in the biosynthesis of MUFA [46]; DGAT-2 catalyzes the final reaction in the synthesis of TGs [47] and sterol regulatory element binding protein 1 is involved in activation of lipogenic genes in the liver [48].

**FIGURE 6:**
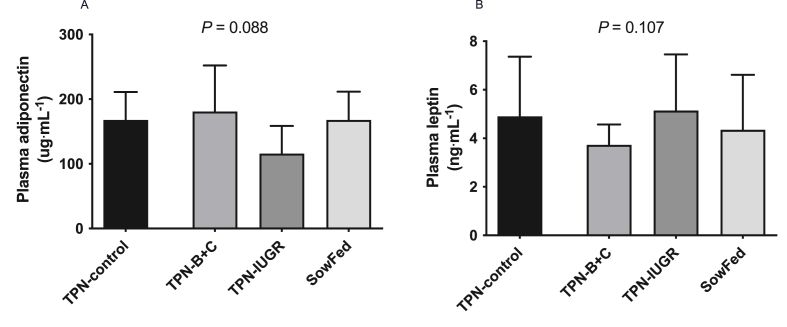
Plasma (A) adiponectin and (B) leptin concentration of adult Yucatan miniature pigs (9 mo) fed 4 experimental diets; values are means ± SD, *n* = 7–8. Data were analyzed using 1-way ANOVA with Dunnett’s post hoc test comparing to TPN-control. ANOVA, analysis of variance; SowFed, suckled; TPN, total parenteral nutrition; TPN – B + C, total parenteral nutrition with betaine and creatine; TPN-control, total parenteral nutrition control diet; TPN-IUGR, intrauterine growth-restricted piglets fed total parenteral nutrition control diet.

Plasma concentrations of adiponectin (*P* = 0.088) and leptin (*P* = 0.107) at 9 mo were not different between the experimental groups (Figure 6).

### Plasma amino acid concentrations

Plasma isoleucine, leucine, and tryptophan have been shown to be greater during CVD [49]. Plasma amino acid profiles at 9 mo were not different between the experimental groups (Table 4); however, isoleucine, leucine, and tryptophan tended (*P* = 0.083, 0.141, 0.084, respectively) to be higher in TPN-IUGR pigs compared to TPN-control pigs.

**TABLE 4:** Plasma amino acid concentrations (*μ*mol‧L^–1^) of Yucatan miniature pigs at 9 mo fed 4 experimental diets; TPN-control, TPN-B+C, TPN-IUGR, and SowFed during the neonatal period.

	TPN-control	TPN – B + C	TPN-IUGR	SowFed	*P* value
**Amino acids related to methionine metabolism**					
Glycine	642.0 ± 143.6	601.3 ± 183.9	521.6 ± 41.7	681.8 ± 144.7	0.218
Serine	111.0 ± 23.0	90.4 ± 22.6	96.8 ± 16.9	103.4 ± 20.2	0.303
Methionine	8.7 ± 2.6	7.7 ± 1.3	10.1 ± 3.5	9.4 ± 3.3	0.498
Taurine	109.3 ± 31.1	118.3 ± 82.6	128.7 ± 53.4	107.2 ± 33.9	0.847
**Indispensable amino acids**					
Histidine	41.5 ± 12.7	41.3 ± 8.5	46.7 ± 18.5	43.5 ± 8.2	0.861
Isoleucine	104.6 ± 15.0	111.6 ± 15.6	119.3 ± 17.9	84.9 ± 40.2	0.083
Leucine	184.9 ± 26.8	195.7 ± 19.3	214.3 ± 42.1	176.1 ± 29.7	0.141
Lysine	231.5 ± 55.5	213.2 ± 47.5	221.7 ± 24.8	189.3 ± 34.7	0.312
Phenylalanine	65.4 ± 8.2	64.4 ± 6.4	57.4 ± 26.5	60.8 ± 6.3	0.867
Threonine	118. ± 40.8	96.2 ± 31.3	127.7 ± 54.7	83.6 ± 8.7	0.096
Tryptophan	82.9 ± 31.1	83.7 ± 43.3	93.4 ± 45.7	87.5 ± 23.6	0.084
Valine	256.6 ± 41.4	272.8 ± 39.2	310.8 ± 96.6	245.3 ± 51.1	0.256
**Dispensable amino acid**s					
Alanine	57.7 ± 18.4	59.6 ± 10.9	55.6 ± 14.2	51.6 ± 10.0	0.393
Hydroxyproline	36.4 ± 7.2	32.5 ± 11.5	37.3 ± 12.8	37.2 ± 9.6	0.819
Tyrosine	72.1 ± 9.4	71.5 ± 12.7	75.2 ± 10.1	64.9 ± 12.0	0.394
Ornithine	131.7 ± 35.8	112.8 ± 40.9	100.0 ± 44.4	114.0 ± 33.4	0.476
Proline	135.1 ± 38.3	144.2 ± 43.7	128.8 ± 22.1	141.0 ± 34.2	0.878
Aspartate	6.7 ± 2.2	5.5 ± 1.4	7.5 ± 2.0	7.9 ± 3.0	0.325
Arginine	57.7 ± 18.4	59.6 ± 10.9	55.6 ± 14.2	51.6 ± 10.0	0.759
Glutamate	13.1 ± 9.5	13.2 ± 6.6	21.3 ± 10.1	12.1 ± 6.9	0.733
Glutamine	207.3 ± 76.0	200.1 ± 76.6	191.7 ± 24.9	295.5 ± 122.9	0.111
Asparagine	28.3 ± 6.6	21.7 ± 8.5	27.8 ± 4.4	28.9 ± 7.5	0.259
Citrulline	24.0 ± 9.3	27.3 ± 8.9	34.0 ± 15.4	29.0 ± 11.0	0.458

Values are means ± SD, *n* = 6–8.

One-way ANOVA with Dunnett’s post hoc test comparing to TPN-control.

Abbreviations: ANOVA, analysis of variance; SowFed, suckled; TPN, total parenteral nutrition; TPN – B + C, total parenteral nutrition with betaine and creatine; TPN-control, total parenteral nutrition control diet; TPN-IUGR, intrauterine growth-restricted piglets fed total parenteral nutrition control diet.

Some amino acids (glycine, serine, tryptophan, alanine, tyrosine, proline, aspartate, and asparagine) were greater in the SowFed compared to TPN-control piglets at 21 d of age (Table 5). These differences in plasma amino acid concentrations are related to differences in diet (TPN compared with sow milk) and feeding mode. However, adding B + C to TPN or being IUGR did not affect plasma amino acid profiles during the TPN phase.

**TABLE 5:** Plasma amino acid concentrations (*μ*mol‧L^–1^) of Yucatan miniature pigs at 21 d fed 4 experimental diets; TPN-control, TPN-B+C, TPN-IUGR, and SowFed during the neonatal period.

	TPN-control	TPN – B + C	TPN-IUGR	SowFed	*P* value
**Amino acids related to methionine metabolism**					
Glycine	209.3 ± 53.2	190.4 ± 27.5	226.9 ± 62.8	332.0 ± 121.9∗	0.003
Serine	621.2 ± 120.1	574.6 ± 178.0	689.4 ± 152.6	336.3 ± 136.2∗	0.0004
Methionine	29.1 ± 13.1	25.1 ± 9.1	28.8 ± 12.8	20.1 ± 11.8	0.426
Taurine	138.9 ± 35.3	123.1 ± 28.6	129.6 ± 33.4	153.7 ± 54.0	0.468
**Indispensable amino acids**					
Histidine	89.9 ± 52.7	119.3 ± 65.1	135.4 ± 86.2	93.0 ± 39.2	0.429
Isoleucine	197.8 ± 54.3	218.3 ± 47.4	184.4 ± 55.0	177.1 ± 47.5	0.420
Leucine	351.8 ± 124.7	387.0 ± 83.0	350.8 ± 133.9	253.7 ± 106.7	0.169
Lysine	240.5 ± 66.3	305.0 ± 116.4	372.2 ± 190.0	222.3 ± 124.8	0.136
Phenylalanine	194.5 ± 54.9	202.1 ± 35.9	178.7 ± 39.6	253.2 ± 121.9	0.179
Threonine	152.1 ± 44.8	163.8 ± 48.8	187.6 ± 64.1	160.5 ± 62.9	0.575
Tryptophan	288.1 ± 44.9	263.7 ± 119.1	306.9 ± 63.4	139.7∗ ± 43.0	0.001
Valine	421.5 ± 61.6	491.3 ± 62.0	358.0 ± 102.3	431.9 ± 156.0	0.068
**Dispensable amino acids**					
Alanine	437.0 ± 33.0	448.1 ± 120.8	453.9 ± 108.4	287.8 ± 85.4∗	0.006
Hydroxyproline	119.2 ± 31.7	122.6 ± 48.4	155.4 ± 44.3	139.9 ± 60.3	0.341
Tyrosine	82.1 ± 26.7	132.4 ± 64.7	123.2 ± 74.8	177.2 ± 49.0∗	0.044
Ornithine	137.1 ± 34.4	118.3 ± 34.9	119.3 ± 42.7	150.9 ± 70.2	0.479
Proline	1023.0 ± 166.5	1063.0 ± 93.4	942.7 ± 240.5	734.0 ± 287.3∗	0.035
Aspartate	108.0 ± 50.0	78.2 ± 26.3	78.0 ± 29.8	50.6 ± 13.8∗	0.043
Arginine	157.0 ± 46.2	184.3 ± 53.1	145.3 ± 71.4	106.5 ± 61.3	0.112
Glutamate	246.9 ± 97.0	206.5 ± 65.5	219.7 ± 79.5	165.9 ± 53.2	0.249
Glutamine	54.4 ± 32.6	75.7 ± 56.3	34.7 ± 34.0	54.1 ± 29.0	0.232
Asparagine	1405.0 ± 242.0	1304.0 ± 489.1	1658.0 ± 361.8	829.6 ± 297.0∗	0.0008
Citrulline	111.3 ± 57.7	103.2 ± 52.7	129.9 ± 69.9	162.7 ± 51.0	0.241

Values are means ± SD, *n* = 7–8.

Abbreviations: ANOVA, analysis of variance; SowFed, suckled; TPN, total parenteral nutrition; TPN – B + C, total parenteral nutrition with betaine and creatine; TPN-control, total parenteral nutrition control diet; TPN-IUGR, intrauterine growth-restricted piglets fed total parenteral nutrition control diet.

∗*P* < 0.05; 1-way ANOVA with Dunnett’s post hoc test comparing to TPN-control.

### Plasma total bilirubin concentrations

**FIGURE 7:**
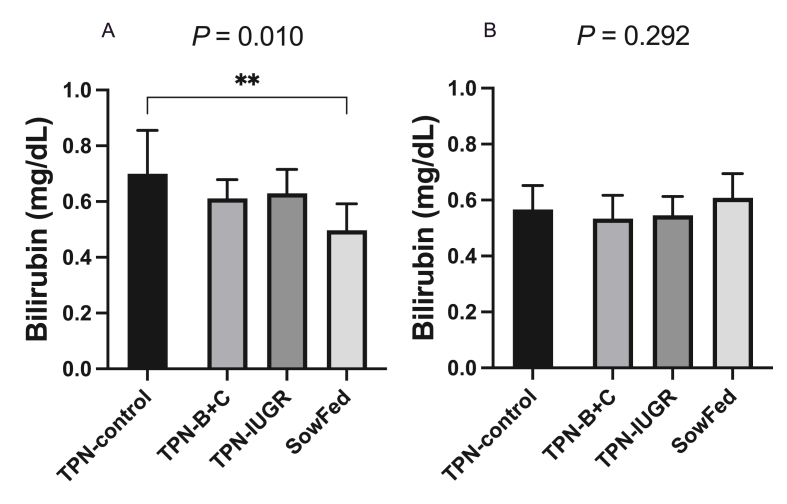
Plasma total bilirubin concentrations (A) at 21d and (B) at 9 mo of age in Yucatan miniature pigs fed 4 experimental diets. Values are means ± SD, *n* = 7–8. Data were analyzed using 1-way ANOVA with Dunnett’s post hoc test comparing to TPN-control. ANOVA, analysis of variance; SowFed, suckled; TPN, total parenteral nutrition; TPN – B + C, total parenteral nutrition with betaine and creatine; TPN-control, total parenteral nutrition control diet; TPN-IUGR, intrauterine growth-restricted piglets fed total parenteral nutrition control diet.

Plasma total bilirubin concentrations, a marker of parenteral nutrition-associated liver disease, were higher in TPN-control, compared to SowFed piglets at 21 d old *P* = 0.010) (Figure 7). However, by 9 mo old, total bilirubin was not different among adult pigs (*P* = 0.29).

### Correlation analyses

Correlation analyses between subcutaneous fat thickness with growth parameters at various stages of development (body weight, body weight gain, and body measurements) and lipid parameters indicate that subcutaneous fat thickness is indicative of obesity and dyslipidemia (Supplemental Figures 1 and 2). Numerous indicators were correlated, although others did not show significant differences.

### Plasma methyl-related metabolites

Plasma concentrations of betaine and DMG, the product of betaine-homocysteine S-methyltransferase activity, were higher in TPN – B + C pigs than in TPN-control in the TPN phase (21 d) when the TPN diet was supplemented with betaine (TPN – B + C) (Table 6). However, plasma DMG concentrations were also within the same range as the clinical reference group (SowFed). These data suggest that increased DMG concentrations in the B + C supplemented group in the piglet stage are due to supplemented betaine to TPN, and the resulting DMG concentrations represent normal levels. Creatine concentrations were not significantly different in the TPN – B + C group compared to the TPN-control group in piglets, even though creatine was supplemented in the TPN – B + C diet. Creatine may have been extracted from plasma rapidly by the piglets, as they are in the accelerated growth and development stage.

**TABLE 6:** Plasma methyl-related metabolites in TPN-control, TPN-B+C, TPN-IUGR, and SowFed Yucatan miniature piglets.

	Age	TPN-control	TPN – B + C	TPN-IUGR	SowFed
**Plasma**					
Homocysteine (*μ*mol‧ L^–1^)	21 d	23.8 ± 3.4	18.7 ± 4.8	46.2 ± 20.7∗∗	25.6 ± 10.2
Dimethylglycine (*μ*mol‧ L^–1^)	21 d	5.3 ± 2.3	18.6 ± 5.5∗∗∗	4.4 ± 3.3	15.5 ± 3.3∗∗∗
Betaine (*μ*mol‧ L^–1^)	21 d	64.2 ± 16.8	358.4 ± 90.5∗∗∗	35.2 ± 20.3	44.2 ± 11.4
Choline (*μ*mol‧ L^–1^)	21 d	8.7 ± 1.7	11.8 ± 4.0	5.9 ± 2.2	8.0 ± 2.7
Creatine (*μ*mol‧ L^–1^)	21 d	277.4 ± 56.7	241.4 ± 58.9	340.9 ± 79.9	188.5 ± 96.9
Homocysteine (*μ*mol‧ L^–1^)	9 mo	35.2 ± 5.1	37.2 ± 7.1	34.6 ± 5.3	35.7 ± 4.9
Dimethylglycine (*μ*mol‧ L^–1^)	9 mo	1.40 ± 0.3	1.5 ± 0.3	2.3 ± 0.8∗	1.5 ± 0.4
Betaine (*μ*mol‧ L^–1^)	9 mo	105.8 ± 26.7	117.9 ± 43.6	114.4 ± 32.9	129.4 ± 44.5
Choline (*μ*mol‧ L^–1^)	9 mo	6.4 ± 1.9	5.8 ± 1.8	6.9 ± 1.8	5.5 ± 1.2
Creatine (*μ*mol‧ L^–1^)	9 mo	300.7 ± 94.8	290.9 ± 72.3	340.7 *±* 88.3	327.1 ± 76.3

Values are means ± SD, *n* = 7–8.

Abbreviations: ANOVA, analysis of variance; SowFed, suckled; TPN, total parenteral nutrition; TPN – B + C, total parenteral nutrition with betaine and creatine; TPN-control, total parenteral nutrition control diet; TPN-IUGR, intrauterine growth-restricted piglets fed total parenteral nutrition control diet.

∗*P* < 0.05, ∗∗*P* < 0.001, ∗∗∗*P* < 0.0001; 1-way ANOVA with Dunnett’s post hoc test comparing to TPN-control.

## Discussion

TPN support is a non-normal lifesaving nutritional regimen and has become an integral part of the medical management of preterm and IUGR neonates. Approximately 9% of births in Canada are preterm, and 8–10% are IUGR [50]. These infants constitute 35–50% of admissions to neonatal intensive care units, cost the health care system 10–20 times more than a healthy infant, and account for 75% of prenatal deaths [51,52]. Studies have demonstrated that although TPN is a lifesaving feeding method, it has short-term drawbacks, such as increased adiposity, hepatic steatosis, and inflammation [5,[53], [54], [55]]. When TPN is administered during the critical window of development in the neonatal period, it may have long-term adverse effects; however, nutritional requirements and interactions during the feeding regimen and their impact on long-term metabolic diseases are not well understood. In addition, research has demonstrated that neonates who are born IUGR are also susceptible to developing catch-up growth, increased adiposity, and impaired lipid metabolism as young adults [25]. Consequently, we hypothesized that metabolic alterations and increased adiposity resulting from early TPN feeding would persist into adulthood and that feeding TPN to IUGR neonates would exacerbate the effects of obesity and dyslipidemia in later life.

We demonstrated that TPN-fed piglets with a normal birth weight maintained a similar body weight as those fed sow milk throughout this early feeding phase. Although IUGR piglets grew in parallel to the TPN-control pigs while receiving TPN, they could not catch-up in body weight by the end of the TPN feeding phase. This lack of catch-up growth during TPN feeding could be due to the restricted supply of TPN solution relative to their body weight (restricted feeding) during this phase. Therefore, IUGR piglets remained underweight at the end of the TPN feeding phase (21 d of age). Following TPN, IUGR pigs appeared to experience a brief growth surge between 21 d and 28 d of age when they were adapted to an ad libitum grower diet. Because each pig adapts to the grower’s diet differently, they were not feed-restricted, and it is possible that IUGR pigs had a higher feed intake, facilitating catch-up growth during this period; however, we did not monitor intakes throughout the acclimation period. Once the pigs were adapted to the grower diet, they were given a restricted diet for the 9-mo grow-out phase. In the current study, although intakes were restricted, significant catch-up growth was recorded in IUGR pigs between 1–4 mo and 4–6 mo, as demonstrated by considerably enhanced body growth rates. In another grow-out trial from our laboratory, IUGR pigs who were given ad libitum consumption after weaning had a comparable catch-up growth spurt around the same age. Both studies exhibited a resurgence of growth postweaning in IUGR pigs. Remarkably, the body weights of all pigs at the end of the current study were not different, although IUGR pigs had increased subcutaneous fat thickness. These data suggest that the overall catch-up “growth” of TPN-fed IUGR pigs included a greater accumulation of adipose in addition to lean mass. Significant positive correlations between body growth rates (Figure 7: A, B, C, D, and E) and body weights to subcutaneous fat thickness further demonstrate that IUGR pigs developed higher adiposity by the end of the study.

Based on the findings of the present investigation, it is evident that IUGR pigs became more obese as young adults. In the event of obesity, excess circulating lipid will lead to increased free fatty acids in the plasma. Continuous exposure of tissues to free fatty acids will lead to the accumulation of TG in tissues that do not normally store excess fat (i.e., ectopic fat deposition) [56]. We demonstrated that TPN-IUGR pigs had higher ectopic fat accumulation in the liver and skeletal muscles, further predisposing them to adverse metabolic effects. Because the liver serves as a metabolic nexus for various tissues, including skeletal muscle and adipose tissue [57], accumulation of TG in the liver could be a key player in obesity and dyslipidemia [58]. To understand whether increased TG in the liver is due to increased lipogenesis and whether lipogenic genes have been programmed and differentially expressed in the adult IUGR pigs due to early TPN feeding, we measured the relative expression of lipogenic genes, including rate-limiting genes responsible for lipogenesis (ACC-1 and FASN) and TG synthesis (DGAT-2). However, we found that the relative lipogenic gene expressions were not different between TPN-IUGR and TPN-control pigs. Therefore, the TG accumulation in the liver of IUGR pigs could be due to delayed TG clearance via lipoprotein lipase. However, we could not measure total lipoprotein lipase activity in the circulation, as we did not inject heparin at necropsy, which would have compromised other parameters measured in the study. From another perspective, the higher plasma total bilirubin concentrations in the TPN-control group compared to the SowFed group is likely due to parenteral nutrition-associated liver disease with liver steatosis [59] at 21 d. However, the absence of differences in total bilirubin concentrations in adult pigs at 9 mo indicates that the metabolic effects of liver steatosis due to TPN feeding resolved after cessation of TPN and the liver steatosis in the TPN-IUGR adult pigs is likely attributable to gene programming effects, rather than persistent parenteral nutrition-associated liver disease.

FASN catalyzes fatty acid synthesis, synthesizing a long-chain saturated fatty acid, palmitate, from acetyl-CoA and malonyl-CoA. Evidence shows that TPN feeding influences the expression of genes involved in fat metabolism in the liver [60]. Moreover, the gene for FASN has been demonstrated to be epigenetically controlled [61]. Hence, the lower relative expression of FASN in TPN-control pigs compared to SowFed pigs could be attributed to the effects of TPN feeding on FASN expression during the neonatal period, which persisted into adulthood. However, we did not measure the expression of genes in neonates, so this expression change could also be due to other TPN-induced changes in lipid metabolism. Adult TPN-control pigs also had higher lipid peroxidation markers compared to SowFed pigs. These data suggest that higher oxidative stress due to neonatal TPN feeding may have persisted into adulthood, likely via changes in the epigenetics of genes related to antioxidant defense systems. The oxidative stress induced by TPN feeding during the first week of life is suspected to re-program energy metabolism in the liver [62]. Generation of by-products of lipid peroxidation is an inherent drawback in using TPN, which can increase oxidative stress [63], and reports have demonstrated novel associations between DNA methylation and oxidative stress [64,65] because IUGR piglets have lower global methylation.

Obesity-related changes in adipocytes stimulate lipolysis and release fatty acids into the circulation [66], as indicated by an increase in NEFA concentrations in the blood of 9-mo-old obese TPN-IUGR pigs. Increased NEFA infiltration into the liver of TPN-IUGR pigs may also contribute to their elevated TG concentrations. The increased hepatic influx of free fatty acids may have resulted in increased VLDL production, as indicated by higher concentrations of VLDL-TG and non-HDL cholesterol after fasting. Therefore, higher adiposity was associated with the development of dyslipidemia in TPN-IUGR pigs. The positive correlation between subcutaneous fat thickness and plasma non-HDL cholesterol and LDL cholesterol indicates that obesity and dyslipidemia are interconnected metabolic disorders. Adiponectin is a cardioprotective hormone that decreases as oxidative stress and inflammation increase, which is common in obese individuals with low-grade inflammation. Although adiponectin concentrations tended to be lower in TPN-IUGR pigs, this biomarker of metabolic syndrome was not different. It is possible that not all biomarkers of metabolic syndrome are programmed by early nutrition. However, it should also be noted that our biomarker measurements were measured in young “adulthood” for pigs. The lack of overt metabolic syndrome is not surprising, and these markers are expected to worsen with age as metabolic syndrome develops. The argument that pigs may be too young to exhibit fasting metabolic syndrome biomarkers is also supported by nonsignificant plasma fasting glucose and insulin concentrations in experimental groups compared to the TPN-control group. Nevertheless, the development of early biomarkers of metabolic syndrome in young adulthood is profound when the only intervention in this experiment was changes in neonatal feeding.

Myocardial TG within cardiomyocytes constitutes a critical fatty acid and energy reserve for the heart [67]. Metabolism of cardiac TG is highly dynamic [68], and it ensures a continuous fatty acid supply for mitochondrial oxidation, independent of short-term fluctuations in plasma fatty acid availability. Because intracellular TG hydrolysis contributes significantly to the generation of ATP necessary for contractile function [69,70], TG reserves play a critical role in regulating cardiac function, and reduced cardiac TG content is associated with cardiac dysfunction [67,71]. Therefore, it has been suggested that greater storage of TG in the cardiac muscle, to some extent, is considered to have a cardioprotective effect. Diverting infiltrating NEFA into TG in the cardiac muscle prevents the cardiac cells from lipotoxicity effects of accumulated fatty acids [72] Thus, reduced cardiac TG reserves in the TPN-control pigs in the current study suggest that they may not have readily available energy stores for energy-demanding cardiac cells, whereas increased cardiac TG content in the TPN – B + C group compared to TPN-control group suggests a complementary effect on cardiac contractility and may likely have contributed to reduced hypertension parameters [73]. Interestingly, cellular NEFA concentration in cardiac cells was maintained at the same level, regardless of the treatment groups, preventing cardiac cells from being exposed to their lipotoxicity effects.

TPN-IUGR pigs are anticipated to shift their amino acid profile toward the phenotype associated with obesity and CVD as their obesity develops with age. After 9 mo, there were no significant differences between the fasting amino acid profiles of TPN-IUGR and TPN-control pigs; however, there were trends toward higher concentrations of isoleucine and leucine in adult TPN-IUGR pigs. Higher plasma concentrations of these amino acids have been associated with an increased risk of CVD [74]. Recent research demonstrates that plasma amino acid profiles are altered in obese individuals, suggesting a correlation between fat accumulation and plasma amino acid profiles [75,76]. Positive correlations of tryptophan, aspartate, alanine, leucine, isoleucine, and tyrosine at 9 mo with obesity-related parameters in the current study (TG concentrations in skeletal muscle, 1–4-mo growth rate, crown-to-rump length, chest girth, and abdominal circumference) suggest that differences in amino acids will become more pronounced as TPN-IUGR pigs’ obesity increases with age.

Given the extensive nature of these long-term studies and the low frequency of IUGR piglets born in our herd, we, unfortunately, did not include a group of IUGR piglets fed TPN supplemented with B + C. However, because we observed significant programming of obesity/lipid outcomes in IUGR pigs fed early TPN, the next logical experiment would be to add betaine and creatine to early TPN-fed to IUGR piglets to determine if B + C could prevent these outcomes. Moreover, additional analyses are warranted to determine if epigenetics is involved in the observed programming of obesity and lipid metabolism.

In conclusion, overall, these findings imply that feeding TPN to neonates did not cause overt obesity in later life, and adding betaine and creatine to TPN also did not affect obesity in later life. However, IUGR did lead to obesity development, which exacerbated the programmed effects of dyslipidemia in adulthood. Our findings will aid in comprehending the long-term programming effects of TPN feeding to IUGR neonates on obesity development so that new nutritional interventions can be designed to reduce the programming risk of adulthood obesity and related metabolic disorders.

## Author contributions

The authors’ responsibilities were as follows– RFB, JAB: designed the research; L-AH: conducted initial animal work; RSR: conducted the study, analyzed the data, and wrote the first draft; RSR, RFB: interpreted the results, wrote the paper and had primary responsibility for the final content; and all authors: read and approved the final manuscript.

## Data availability

Data are available on request.

## Funding

This project was supported by the operating grants to RFB from the Canadian Institutes of Health Research.

## Conflict of interest

RFB is an editorial board member of The Journal of Nutrition and played no role in the Journal’s evaluation of the manuscript. All other authors report no conflicts of interest.
