# Supplementary material for: Intrauterine Growth-Restricted Female Yucatan Miniature Pig Neonates Fed Parenteral Nutrition Exhibit Early Catch-Up Growth Leading to Obesity and Ectopic Fat Deposition in Adulthood
Source: J Nutr. 2025 May 27;155(8):2653–67. doi: 10.1016/j.tjnut.2025.05.031 (PMC12405908; doi:10.1016/j.tjnut.2025.05.031)
Supplement: Multimedia component 1 [file mmc1.docx]

**Supplemental Table 1**: Amino acid profile of TPN diets

| **Amino acid profile** | **TPN-control diet (g‧L^-1^)** | | **TPN B+C diet (g‧L^-1^)** |
| --- | --- | --- | --- |
| Alanine | 5.89 | 5.89 | |
| Arginine | 3.65 | 3.65 | |
| Aspartic Acid | 3.32 | 3.32 | |
| Cysteine | 0.76 | 0.76 | |
| Glutamic Acid | 5.72 | 5.72 | |
| Glycine | 1.47 | 1.47 | |
| Histidine | 1.69 | 1.69 | |
| Isoleucine | 2.51 | 2.51 | |
| Leucine | 5.67 | 5.67 | |
| Lysine hydrochloride | 5.58 | 5.58 | |
| Methionine | 1.04 | 1.04 | |
| Phenylalanine | 3.00 | 3.00 | |
| Proline | 4.52 | 4.52 | |
| Serine | 3.11 | 3.11 | |
| Taurine | 0.27 | 0.27 | |
| Tryptophan | 1.14 | 1.14 | |
| Tyrosine | 0.44 | 0.44 | |
| Threonine | 2.23 | 2.23 | |
| Valine | 2.89 | 2.89 | |
| Betaine hydrochloride | 0 | 1.29 | |
| Creatine monohydrate | 0 | 0.57 | |

TPN, Total parenteral nutrition; TPN-control, TPN control diet; TPN-B+C, TPN control diet supplemented with betaine and creatine.

**Supplemental Table 2:** Composition of the grower diet (12.1 MJ digestible energy‧kg^-1^ and 154 g protein‧kg^-1^)

| ***Energy (% total energy)*** |  |
| --- | --- |
| Carbohydrate | 67 |
| Fat | 12 |
| Protein | 21 |
| ***Ingredients (g‧kg^-1^ dry matter)*** |  |
| Wheat shorts | 400.5 |
| Canola | 49.0 |
| Meat meal | 19.0 |
| Limestone | 13.0 |
| Corn gluten feed | 40.0 |
| Ground barley | 297.0 |
| Oats | 175.0 |
| Vitamin mix* | 0.8 |
| Mineral mix** | 1.0 |
| Sodium Chloride | 4.7 |

*Each kg of grower diet contained a vitamin mix of vitamin A, 4900000 IU; vitamin D3, 580000 IU; vitamin E, 22000 IU; vitamin K, 1.1 g; biotin 67 mg; folacin, 622 mg; niacin, 18 g; pantothenic acid, 10 g; riboflavin, 3.1 g; thiamin, 833mg; vitamin B6, 833mg and vitamin B12, 11 µg.

**Each kg of grower diet contained a mineral mix of copper, 8.3 g; iodine, 0.3 g; iron, 55.5 g; manganese, 22.2 g; selenium, 0.2 mg; zinc, 55.5 g. Calcium, phosphorus, sodium, chloride, magnesium, and potassium were provided through limestone, which contained that mineral on a % basis, 35.84, 0.01, 0.06, 0.02, 2.06, 0.11 respectively.

**Supplemental Table 3:** Primer pairs used for real-time PCR.

| **Gene** | **Gene symbol** | **Forward** | **Reverse** | **Gene Bank accession no** | **References** |
| --- | --- | --- | --- | --- | --- |
| Acetyl co A carboxylase | ACC-1 | ATA CCC GTG GGA GTA GTT GC | GCG GTT GAA GTC CTT GAT GG | NM_001114269.1 | Xing K. *et al*., 2014 |
| Diacylglycerol acyltransferase 2 | DGAT-2 | GCA GGT GAT CTT TGA GGA GG | GCT TGG AGT AGG GCA TGA G | NM_001160080.1 | Cui J.X., *et al*., 2011 |
| Fatty acid synthase | FASN | ACA CCT TCG TGC TGG CCT AC | ATG TCG GTG AAC TGC TGC AC | NM_001099930 | Madeira M.S., *et al*., 2016 |
| Sterol co- A desaturase | SCD | AGC CGA GAA GCT GGT GAT GT | GAA GAA AGG TGG CGA CGA AC | NM_213781 | Madeira M.S., *et al*., 2016 |
| Sterol regulatory element binding protein 1c | SREBP-1C | AAG CGG ACG GCT CAC AA | GCA AGA CGG CGG ATT TAT T | NM_214157.1 | Qiu Y. *et al*., 2017 |
| Carnitine palmitoyl transferase 1 | CPT1 | ACA AGC CAT AGT CTT AAC GAA A | GCC AGT CCA GGA TAA CAA A | NM_001129805 | He J., *et al*., 2011 |
| Fatty acid translocase | CD36 | GCA CAG AAA AAG TTG TCT CCA AAA AT | ATG TAC ACA GGT TTT CCT TCT TTG C | NM_001044622.1 | De Tonnac A. *et al*., 2016 |
| Glyceraldehyde-3-phosphate dehydrogenase | GAPDH | ATCCTGGGCTACACTGAGGA | TGTCGTACCAGGAAATGAGCT | DQ845171 | Park S., *et al*., 2015 |
| Beta actin | β actin | CAC GCC ATC CTG CGT CTG GA | AGC ACC GTG TTG GCG TAG AG | NM_001206359.1 | Nygard A. *et al.,* 2007 |

Xing K, Zhu F, Zhai L, Liu H, Wang Z, Hou Z, Wang C. The liver transcriptome of two full-sibling Songliao black pigs with extreme differences in backfat thickness. J Anim Sci Biotechnol. 2014;5(1):32.

Cui JX, Zeng YQ, Wang H, Chen W, Du JF, Chen QM, et al. The effects of DGAT1 and DGAT2 mRNA expression on fat deposition in fatty and lean breeds of pig. Livest Sci. 2011;140:292–6.

Madeira MS, Pires VM, Alfaia CM, Lopes PA, Martins SV, Pinto RM, Prates JA. Restriction of dietary protein does not promote hepatic lipogenesis in lean or fatty pigs. Br J Nutr. 2016;115(8):1339-51.

Qiu YQ, Yang XF, Ma XY, Xiong YX, Tian ZM, Fan QL, et al. CIDE gene expression in adipose tissue, liver, and skeletal muscle from obese and lean pigs. J Zhejiang Univ Sci B. 2017;18(6):492-500.

He J, Chen D, Zhang K, Yu B. A high-amylopectin diet caused hepatic steatosis associated with more lipogenic enzymes and increased serum insulin concentration. Br J Nutr. 2011;106(10):1470-5.

De Tonnac A, Labussière E, Vincent A, Mourot J. Effect of α-linolenic acid and DHA intake on lipogenesis and gene expression involved in fatty acid metabolism in growing-finishing pigs. Br J Nutr. 2016 Jul;116(1):7-18.

Park J, Mabuchi M, Sharma A. Multiplexed Fluorescent Immunodetection Using Low Autofluorescence Immobilon®-FL Membrane. Methods Mol Biol. 2015;1314:195-205.

Nygard AB, Jørgensen CB, Cirera S, Fredholm M. Selection of reference genes for gene expression studies in pig tissues using SYBR green qPCR. BMC Mol Biol. 2007 Aug 15;8:67.

**Supplemental Figure 1**

a)

b)

c)

d)

e)

f)

g)

h) )

**Supplemental Figure 1:** (A) Correlations between subcutaneous fat thickness and (A) body weight at 4 mo, (B) body growth rate at 1-4 mo, (C) body growth rate at 4-6 mo, (D) body weight at 6 mo, (E) body growth rate at 6-8 mo, (F) body weight at 8 mo, (G) abdominal girth at necropsy, (H) crown to rump length at necropsy in Yucatan miniature pigs fed four experimental diets in the neonatal period.

Data are given as mean ± SD; n = 7-8. Data were analyzed using Pearson correlation analysis. Each symbol represents an individual pig. TPN-Control (●); TPN-B+C (▼); TPN-IUGR (○), SowFed (◊). TPN, Total parenteral nutrition; TPN-control, TPN control diet; TPN-B+C, TPN control diet supplemented with betaine and creatine; TPN-IUGR, IUGR piglets fed TPN-control diet; SowFed, suckled.

**Supplemental Figure 2**

a)


**Supplemental Figure 2:** Correlations between subcutaneous fat thickness and (A) plasma LDL-cholesterol at necropsy, (B) plasma non-LDL-cholesterol at necropsy in Yucatan miniature pigs fed four experimental diets in the neonatal period. Data are given as mean ± SD; n = 7-8 pigs per group. Data were analyzed using Pearson correlation analysis. Each symbol represents an individual pig. TPN-Control (●); TPN-B+C (▼); TPN-IUGR (○), SowFed (◊). TPN, Total parenteral nutrition; TPN-control, TPN control diet; TPN-B+C, TPN control diet supplemented with betaine and creatine; TPN-IUGR, IUGR piglets fed TPN-control diet; SowFed, suckled.

b)
